# Precocious puberty: The clinical profile and the etiological classification of children presented at a tertiary care children’s hospital

**DOI:** 10.12669/pjms.38.4.4816

**Published:** 2022

**Authors:** Sommayya Aftab, Jaida Manzoor, Qaiser Mahmood, Tahir Shaheen

**Affiliations:** 1 Dr. Sommayya Aftab, MBBS, FCPS (Paediatric), MRCPCH (UK). Department of Paediatric Endocrinology and Diabetes, The Children’s Hospital & The Institute of Child Health, Lahore, Pakistan; 2 Dr. Jaida Manzoor, MBBS, FCPS (Paediatric). Department of Paediatric Endocrinology and Diabetes, The Children’s Hospital & The Institute of Child Health, Lahore, Pakistan; 3 Dr. Qaiser Mahmood, MBBS. Department of Radiology, The Children’s Hospital & ICH Lahore, Pakistan; 4 Dr. Tahir Shaheen, MBBS, FCPS (Paediatric). Department of Paediatric Endocrinology and Diabetes, The Children’s Hospital & The Institute of Child Health, Lahore, Pakistan

**Keywords:** Precocious puberty, Central precocious puberty, Peripheral precocious puberty

## Abstract

**Objectives:**

To determine the clinical spectrum and underlying etiologies of children presented with precocious puberty at The Children’s Hospital &The Institute of Child health, Lahore.

**Methods:**

It is a retrospective review of all the children presented with precocious puberty over one year, from January 2015 to December 2015; at the department of Paediatric Endocrinology & Diabetes, The Children’s Hospital & The Institute of Child Health, Lahore.

**Results:**

Total 43 cases of precocious puberty (PP), with 26 females were reported in one year. Central precocious puberty (CPP) constituted 55.8% (24/43) and was found to be more prevalent in female (22/24). In 20/24 cases (83.3%) of central precocious puberty underlying etiology was idiopathic. Peripheral precocious puberty was found in 19/43 cases (44.1%) with male predominance (15/19). Congenital adrenal hyperplasia was the most frequent (12/19) underlying cause of peripheral precocious puberty in our cohort.

**Conclusion:**

Precocious puberty could be a manifestation of underlying serious medical condition. It should be thoroughly evaluated with the aim to diagnose the underlying pathology and to treat them promptly.

## INTRODUCTION

Precocious puberty is defined as precocious manifestation of pubertal development i.e., secondary sexual characters. The consensus about the age to investigate a precocious puberty is before the age of eight years in a girl and 9 years in a boy. The primary concern in precocious puberty is to look for its underlying etiology because precocious puberty could be a clinical manifestation of underlying serious etiology like brain tumour, adrenal or gonadal tumors etc., which needs prompt evaluation and management. However, the secondary concern is rapid bone maturation with early closure of epiphysis leading to short final height.^1^

There are two main underlying mechanisms of precocious puberty. If there is early activation of hypothalamic-pituitary-gonadal (HPG) axis, it is called gonadotropin dependent (central or true) precocious puberty. It’s estimated incidence in American girls is 1 in 5000 –10,000.^2^ However, it is one in 500 among Danish girls, based on national registries over a 9-year period (1993-2001).^3^ Precocious puberty seems sexually dimorphic, being higher in girls than in boys (15-20 girls for every boy).^4^

Second mechanism is increased autonomous production of sex steroids, independent from hypothalamic-pituitary axis activation. Source of these sex steroid are gonads, adrenals, exogenous administration, or ectopic production from tumors. This type of precocious puberty is called gonadotropin independent (peripheral or pseudo) precocious puberty. Peripheral precocious puberty (PPP) has varied etiology including congenital adrenal hyperplasia, virilizing adrenal tumors, gonadal tumors, hypothyroidism, Macune-Albright syndrome and testotoxicosis.^5^

In Pakistan, precocious puberty is a neglected area and little work has been done so far to look at its etiology in our population. The Children’s Hospital & The Institute of Child Health, Lahore is a tertiary care hospital with huge referral of children with pubertal problem in its endocrine department. The purpose of conducting this study was to assess the clinical profile and causes of precocious puberty in our population. Our own data could be a representative of our population and will be helpful in making our local guidelines.

## METHODS

After taking approval by institutional review board (Ref. No 02/CH/ICH, dated 29/03/2017), records of all children with precocious puberty presented to the Department of Paediatric Endocrinology & Diabetes, The Children’s Hospital & ICH, from January 2015 to December 2015 were retrospectively reviewed in detail. Boys less than eight years of age and girls less than nine years of age with appearance of any secondary sexual character were included in this study. Children with atypical genitalia were excluded from this study.

History including age of presentation, duration of symptoms, presenting complains, pattern of secondary sexual character, history of hypothyroidism, any past neurological event (meningitis, brain surgery, brain tumors etc.) and developmental delay were noted. Examination including height, weight, vitals, dysmorphism, pigmentation, tanner staging, complete genital examination, abdominal examination, and neurological examination were retrieved. Hormonal profile including baseline pituitary function test (luteinizing hormone (LH) and follicle stimulating hormone (FSH), thyroid function test, insulin like growth factor-1 (IGF1), adrenocorticotropin hormone (ACTH) and cortisol), testosterone, dehydroepiandrosterone sulfate (DHEASO4), estradiol, and 17-OH progesterone (17 OHP) were noted. GnRH stimulation test was done by giving triptorelin acetate 0.1 mg subcutaneous followed by 20 min and 60 min of LH and FSH in cases where needed. Imaging including bone age by Greulich-Pyle method, ultrasound pelvis and abdomen, and MRI brain or abdomen reviewed thoroughly. All the information were recorded on predesigned proforma. Children with isosexual and concordant pattern of precocious puberty showing pubertal response of gonadotropins either on basal or on GnRH stimulation test were classified as central precocious puberty. All cases of CPP underwent brain imaging (CT/MRI) to look for underlying organic brain pathology. Children with discordant pattern of puberty with prepubertal gonadotropin response on GnRH stimulation test were considered as PPP. Each case peripheral precocious puberty was thoroughly investigated to look for the source of sex steroid hormone production. Children with peripheral precocious puberty and raised 17-OH progesterone (>300 nmol/l) were labeled as congenital adrenal hyperplasia. Peripheral precocious puberty with clinical suspicion of hypothyroidism, having raised TSH (> 10 uIU/ml) and low Free T4(<0.89 ng/dl) were diagnosed as chronic primary hypothyroidism. PPP due to adrenal tumors were confirmed by CT/MRI proven adrenal masses in the presence raised adrenal androgens (testosterone, DHEASO4, and androstenedione). PPP due to gonadal tumors were confirmed by biochemical evidence of excess of testosterone (> 30 ng/dl) or estradiol (> 31 pmol/l) with CT/MRI proven gonadal masses.

Macune- Albright syndrome is defined as precocious puberty due to auto-stimulated ovaries or testis (Ultrasonographical stimulated ovaries or testis with raised estradiol or testosterone and with pre-pubertal or suppress LH (< 2 IU/l) and FSH level (< 2 IU/l) on GnRH stimulation) in presence of hyper pigmented macule and/or fibrous dysplasia. The genetic evaluation of Macune-Albright and testotoxicosis is not available in Pakistan.Data was analyzed by SPSS Version 20. Age of presentation was presented by calculating mean with range. Rest of data was presented by calculating frequencies and percentages.

**Table-I:** Showing underlying etiology of Central Precocious Puberty.

Underlying Etiology	Cases	Percentages
Idiopathic	20	83.33 %
Hydrocephalous	2	8.33 %
Hypothalamic Hamartoma	1	4.16 %
Brain Atrophy (CP)	1	4.16 %

Total Cases	24	100%

## RESULTS

Total 43 cases of precocious puberty, including 24 cases (55.8%) of CPP and 19 cases (44.2%) of PPP, were presented at the Department of Paediatric Endocrinology & Diabetes, The Children’s Hospital & The Institute of Child Health, Lahore. Overall, precocious puberty was more common in females (26/43) than male (17/43) in our study. Among females CPP constituted 84.6% (22/26) and PPP were 15.4% (04/26 cases). In male, PPP was more common (15/17) than CPP (02/17).

Mean age of presentation of precocious puberty was 4.48 + 2.06 years. 19/43 cases (44%) presented between 1 - 5 years of age and 24/43 (56%) between 5-9 years. Six cases (14%) had a family history of precocious puberty. Advanced bone age was found in 40/43 (93%), however, three cases (7%) had delayed bone age due to underlying chronic untreated hypothyroidism. Mean duration of symptoms before presentation was 11.98 ± 9 months.

**Table-II:** Showing underlying etiology of Peripheral Precocious Puberty.

Underlying Etiology	Cases	Percentages
Congenital adrenal hyperplasia	12	63.16 %
Hypothyroidism	3	15.79 %
Wilms tumors	1	5.26 %
Adrenocortical tumor	1	5.26 %
Ovarian tumor	1	5.26 %
Testotoxicosis	1	5.26 %

Total Cases	19	100%

Most frequent clinical presentation in males was pubarche 12/17 (70.6%), followed by increased phallus length 4/17 (23.5%) and tall stature 1/17 (5.9%). In female 11/26 (42.3%) presented with premature breast development, 10/26 (38.5%) with pubarche and 5/26 (19.2%) with menarche.

In female central precocious puberty was the most common type of precocious puberty (22/26) with idiopathic being the most common cause (19/22), followed by hydrocephalous (2/19), and hypothalamic hamartoma (1/19). Four cases of peripheral precocious puberty were reported in females with three having untreated hypothyroidism and one having ovarian tumor as an underlying etiology (Table-III).

**Table-III:** Showing underlying etiologies of different precocious puberty in male and female.

Gender	Classification (n)	Etiology	Number (%)
Male	Central precocious puberty (02)	Idiopathic	01 (5.9%)
		Brain atrophy (CP)	01 (5.9%)
	Peripheral precocious puberty (15)	Congenital Adrenal Hyperplasia	12 (70.5%)
		Wilms tumor	01 (5.9%)
		Adenocarcinoma	01 (5.9%)
		Testotoxicosis	01 (5.9%)
Female	Central precocious puberty (22)
		Idiopathic	19 (73.1%)
		Hydrocephalous	02 (7.7%)
		Hypothalamic hamartoma	01 (3.8%)
	Peripheral precocious puberty (04)	Hypothyroidism	03 (11.6%)
		Ovarian tumor	01 (3.8%)

Among male peripheral precocious puberty was the most common (15/17) type of precocious puberty with congenital adrenal hyperplasia (CAH) being the most common (12/15), followed by virilizing adrenal carcinoma (1/15), wilms tumor (1/15) and testotoxicosis (1/15). There were only two cases of central precocious puberty in male with one having underlying cerebral atrophy (known cerebral palsy) and in other cause was idiopathic (Table-III).

## DISCUSSION

Traditionally, precocious puberty was defined as onset of secondary sexual characters at an age which is 2 to 2.5 standard deviation (SD) below the mean age of onset of puberty for sex.^6^ Consensus is made to workup for precocious puberty if secondary sexual characters start developing before the age of eight year for girls and nine years for boys.^7^-^9^ Younger age of presentation is more concerning and needs thorough evaluation for underlying cause. Certain factors like obesity, family history of precocious puberty, African American ethnic group could be indicators of early presentation of normal precocious puberty.^10^,^11^

Precocious puberty is broadly classified into two main types. Central precocious puberty which is an early maturation of hypothalamic-pituitary-gonadal axis and is characterized by normal sequential appearance of isosexual secondary sexual characters.^4^ Peripheral precocious puberty is caused by excess secretion of sex hormones from the gonads or adrenal, exogenous sources of sex steroids, or ectopic production of gonadotropin from a germ cell tumor. It could be isosexual or heterosexual.^5^ Overall, central precocious puberty is more common than peripheral precocious puberty^4^,^5^ and the same trend was noted in our study where central precocious puberty constituted 55.8% and peripheral precocious puberty constituted 44.2%.

Precocious puberty is reported to be more prevalent in females than males with female to male ratio reported to be 10:1 approximately.^2^ The same pattern of high incidence of precocious puberty in female was reported by Kaplowitz P et al.^12^ and Rohani F et al.^13^, where female represented 87% and 86.5% of precocious puberty, respectively. In our study 60% of total cohort was female with female to male ratio of1.5:1. This ratio is not that high as in other studies which we believe could be under-reporting of precocious puberty in females in our population.

In our study the most common cause of CPP was idiopathic (83.33%), the same trend was reported by Irum et al^14^ and Cisternino et al.^15^ However, 4/24 cases in our cohort were found to have underlying brain pathology called neurogenic CPP. Contrast-enhanced magnetic resonance imaging (MRI) is recommended in CPP in all boys regardless of age and girls presented less than six years of age, even in the absence of clinically evident neurologic abnormalities. Keun et al. reported about 74% of boys with CPP in his study were having brain pathology.^16^ Sena et al reported in meta-analysis that the prevalence of intracranial pathology was 3% in girls with CPP after six years of age, as compared to 25% among those presenting before six years.^17^ Similarly, Stefania et al reported that their data reported that there is no need for brain imaging in girls presenting with CPP after the age of six year.^18^

In our cohort that most common cause of PPP was congenital adrenal hyperplasia (63.16%), followed by hypothyroidism (15.79%), ovarian tumor (5.26%), adrenal tumor (5.26%), wilms tumor (5.2 6%) and testotoxicosis (5.26%). This trend is also observed by Irum et al.^14^ Almost 70.5% cases of PPP in male were congenital adrenal hyperplasia, making it most common cause of PPP in this gender. Precocious puberty due to long standing hypothyroidism called “overlap” or Van Wyk-Grumbach syndrome is a rare entity and reported by many authors.^19^-^21^ It is caused by cross stimulation and activation of FSH receptors by TSH in longstanding and uncontrolled/untreated primary hypothyroidism. In our study 03 females with PPP due to congenital hypothyroidism were reported. They presented with the thelarche, menarche, ovarian masses, and delayed bone age.

Our study is the only study showing the clinical profile of precocious puberty with their underlying etiology in our province – Punjab. Our data support that CPP seems to be more common in female, however, PPP seems to be more common in male. Most of our CPP patient were having normal brain imaging, so we need to have our local guidelines for indication of brain imaging in CPP. In our cohort most of PPP were sequel of delayed presentation of early detectable conditions like CAH and hypothyroidism. Early diagnosis and prompt treatment of these conditions can change their prognosis.

### Limitation of study:

This is a retrospective study and is conducted only over a period of one year. There is a need for prospective study with emphasis of management aspects and long term follow up.

## CONCLUSION

Precocious puberty has varied underlying etiologies. In our population central precocious puberty is more common in female with idiopathic being the most common cause. However, in male peripheral precocious puberty is more prevalent with congenital adrenal hyperplasia being the commonest underlying etiology.

### Authors’ Contribution:

**SA:** Conceived the idea, data collection, Literature search, result processing, writing manuscript. Responsible and accountable for integrity of work.

**JM and QM:** Data collection, literature search, result processing.

**TS:** Literature search, writing manuscript.
